# Dietary acid load modifies the effects of ApoA2–265 T > C polymorphism on lipid profile and serum leptin and ghrelin levels among type 2 diabetic patients

**DOI:** 10.1186/s12902-022-01083-7

**Published:** 2022-07-26

**Authors:** Faezeh Abaj, Zahra Esmaeily, Zeinab Naeini, Masoumeh Rafiee, Fariba Koohdani

**Affiliations:** 1 Department of Community Nutrition, School of Nutritional Sciences and Dietetics, Tehran University of Medical Sciences, Tehran, Iran; 2 Department of Cellular and Molecular Nutrition, School of Nutritional Sciences and Dietetics, Tehran University of Medical Sciences, PO Box: 141556117, Tehran, Iran; 3 Department of Clinical Nutrition, School of Nutrition and Food Science, Isfahan University of Medical Sciences, Isfahan, Iran

**Keywords:** APOA2–265 T > C polymorphism, T2DM, Nutrigenetic, Dietary acid load

## Abstract

This investigation with aimed the effect of APOA2–265 T > C polymorphism and dietary acid load (DAL) as either *potential renal acid load* (*PRAL)* and *net endogenous acid production* (*NEAP*) intake interaction on metabolic markers in type 2 diabetes mellitus (T2DM). In present cross-sectional study, 737 patients with T2DM (290 men and 447 women) were enlisted from diabetes centers in Tehran. The dietary intakes of all participants during the last year was acquired by a validated semi-quantitative food frequency (FFQ) questionnaire. *Polymerase chain reaction (PCR)* was used for *genotyping* the APOA2–265 T > C. Biochemical indises containing leptin, ghrelin, total cholesterol (Bailey et al., J Clin Invest 97:1147–1453, 1996), low-density lipoprotein cholestrol (LDL-C), high-density lipoprotein cholestrol (HDL-C), triglyceride (TG), superoxide dismutase (SOD), high sensitivy C-reactive protein (hs-CRP), total antioxidant capacity (TAC), pentraxin-3 (PTX3), prostaglandin F2α (PGF2α) and interleukin 18 (IL18) were measured by standard method. Atherogenic indices (AIP, AC, CR-I, CR-II) were calculated. The gene-diet interactions were evaluated using an GLM. The frequency overall prevalence of rs5082 genotypes was 63.82 and 36.17% for T-allele and C-allele respectively. TG, Ghrelin, and hs-CRP concentrations were significantly higher among carriers with C allele than TT homozygotes. However, TC/CC genotypes have lower PTX3 than TT homozygotes (*P* < 0.05). C-allele carriers had highest mean of BMI (P_NEAP=_0.04, P_PRAL_ = 0.006), WC (P_NEAP=_0.04, P_PRAL_ = 0.04), TC (P_NEAP=_0.03, P_PRAL_ = 0.01), ghrelin (P_NEAP=_0.01, P_PRAL_ = 0.04), and leptin (P_NEAP=_0.04, P_PRAL_ = 0.03) when placed in top tertiles of NEAP and PRAL.BMI, WC, TC, ghrelin, and leptin levels may be modified in C carriers by decreasing DAL, though, further investigations are required to confirm these findings.

## Introduction

The prevalence rate of diabetes among Iranian adults is rising rapidly which is expected to reach 9.2 million by 2030 [1]. Cardiovascular diseases (CVDs), known as the most common diabetes complication, are responsible for mortality among these patients, approximately 10 times the normal person [2]. Obesity, stress oxidative and dyslipidemia are important risk factors placing diabetic patients at increased risk for CVDs [3, 4]. It is believed that oxidative stress has a vital role in the progression of vascular complications in T2DM. High ROS level in diabetes may be due to reduced Superoxide dismutase (SOD) which is known as an antioxidant enzyme and a major defender against superoxide [5, 6].

Moreover, dyslipidemia is an abnormal condition defined by elevated levels of low density lipoprotein cholesterol (LDL-C), triglycerides (TG) and decreased level of high density lipoprotein cholesterol (HDL-C) is responsible for the appearance of CVDs among patients with Type 2 diabetes mellitus (T2DM) [7].

T2DM and CVDs have been long recognized as complex disorders result by interactions between genetic and environmental factors [8, 9]. One of the main genes involved in increased risk of obesity, T2DM, and CVDs is apolipoprotein A2 (ApoA2) [10]. This gene encodes ApoA2 protein which is the second most abundant protein of HDL-C particles [10, 11]. It seems to impair the reverse transportation and antioxidant function of HDL-C [12] So, increased level of APOA2 promotes the development of atherosclerosis and this is considered to be a major predictor of CVDs [7, 10, 11, 13]. Cardio-metabolic risk factors may vary in different variants of ApoA2 polymorphism among patients with T2DM [7]. APOA2–265 T > C polymorphism (rs5082) is one of the single nucleotide polymorphisms (SNPs) which is related to anthropometric indices, obesity, insulin resistance, and plasma lipids level [7, 10, 11]. Most of studies have found that homozygous individuals for C allele have higher level of central obesity, BMI, and TC [7, 14–16]. They suggested a direct impact of allele-265 T/C on TG and free fatty acids metabolism and revealed that overexpression of apoA II may cause a major increase in the level of apoB containing lipoproteins [17–19].

Findings suggested a strong relationship between APOA2–265 T > C polymorphism and leptin and ghrelin levels as an effective possible mechanism for obesity which reported a high serum ghrelin among CC patients [20, 21]. These two hormones have been recognized to play a major role in appetite regulation and body energy balance [22, 23]. Also, several studies have been shown that leptin and ghrelin levels are strongly affected by dietary intake [24–27].

Contradictory findings in previous researches highlight exploring the interaction between diet and this SNP which may affect on anthropometric indices and cardio metabolic markers [7]. Diet as a key factor may modulate the risk of diabetes and CVDs via interacting with genome [28, 29]. Dietary acid load (DAL) as a nutritional indicator has been considered for its metabolic effects [30]. DAL reflects the acid-forming potential of a diet containing the potential renal acid load (PRAL) and net endogenous acid production (NEAP) estimated by dietary intake [31]. High animal protein intake and excessive intake of artificially sweetened beverages and processed meat and low consumption of fruit and vegetables, known as western dietary pattern, induce a low-grade metabolic acidosis and increase the risk of T2DM [32, 33]*.* It has been suggested a potential relationship between metabolic acidosis status, expressed mainly by PRAL/NEAP, and cardiometabolic abnormalities in diabetic patients [30, 34, 35].

Growing evidence suggests that inflammatory pathways as common pathogenetic mediators for diabetes complications have been developed due to following an unhealthy lifestyle [36]. Pentraxin-3 (PTX3) is a plasma protein involving in chronic inflammation and Interleukin-6 (IL-6) as one of the most common molecules contributes to inflammation may promote endothelial dysfunction and the progression of vascular complication [37]. Moreover, a close relationship has been suggested between dietary pattern and lipid profile [38, 39]. Several studies reported lower HDL-C level in higher tertiles of PRAL/NEAP [40, 41]. Moreover, increased BMI has been observed in those with higher PRAL/NEAP intake [42, 43]. Although, nutrients and foods usually interact with genes, a few studies have investigated gene-diet interactions among individuals with APOA2–265 T > C polymorphism [28, 44]. Previous findings showed a decreased level of IL-18 and hs-CRP in higher polyunsaturated fatty acids (PUFA) intake and increased BMI, LDL-C, LDL/HDL by consuming higher amount of saturated fatty acids (SAFA) among individuals with CC genotype [11, 44, 45]. Since no investigation searched interactions between different dietary patterns and ApoA2 polymorphisms, we aimed to investigate how DAL interacts with APOA2–265 T > C on metabolic markers including TC, LDL-C, HDL-C, TG, ghrelin, leptin, total antioxidant capacity (TAC), SOD, IL-18, PTX3 and prostaglandin F2α (PGF2α) among patients with T2DM.

## Methods and materials

### Study population

This cross-sectional study is a part of a project conducted on 737 patients with T2DM (290 men and 447 women). Participants with fasting blood glucose (FPG) levels of ≥126 mg/dl or were under treatment with medication (oral) were selected randomly from the diabetes centers in Tehran 187 patients were suffering from diabetes for more than 10 years and diabetes duration was less than 10 years among 550 patients. Patients were excluded if they were under 35 or over 65 years old, receiving insulin, intake of vitamin, mineral and herbal supplements, and pregnant or lactating women. The written informed consent was obtained from all the participants. Different information such as age, education, medical family history, medication history, duration of diabetes history was collected using pre-tested questionnaires [11]. All protocols of this study were conducted in accordance with Helsinki Declaration and approved by the Ethics Committee of Tehran University of Medical Sciences (IR.TUMS.VCR.REC.1395.15060). All of the participants completed a written informed consent form before taking part in the study.

### Measurements

Dietary intake was collected using a semi-quantitative food frequency questionnaire (FFQ) (148 food items) through interview by nutrition expert. This questionnaire was validated in Iran [46]. The frequency of daily consumption of each food item for all participants were calculated via U.S. Department of Agriculture and Food Composition Table of Iran. The portion sizes of consumed foods were converted to grams. Data were analyzed by Nutritionist III software (version 7.0, N-Squared Computing).

DAL was estimated by previously established algorithms:

$$\mathrm{PRAL}\ \left(\mathrm{mEq}/\mathrm{day}\right)=0.4888\times \mathrm{protein}\ \left[\mathrm{g}/\mathrm{day}\right]+0.0366\times \mathrm{Phosphorus}\ \left[\mathrm{mg}/\mathrm{day}\right]-0.0205\times \mathrm{Potassium}\ \left[\mathrm{mg}/\mathrm{day}\right]-0.0125\times \mathrm{calcium}\ \left[\mathrm{mg}/\mathrm{day}\right]-0.0263\times \mathrm{magnesium}\ \left[\mathrm{mg}/\mathrm{day}\right].$$

NEAP (mEq/day) = (54.5 × protein [g/day]/potassium [mEq/day]) − 10.2. To estimate the NEAP and PRAL, the nutrients intakes were adjusted for energy intake by residual method [47–49].

Weight, height, and WC were computed in the fasting state and minimal clothing via Seca falcon scales (the nearest 100 g and 0.5 cm, respectively) [50]. BMI was estimated as weight (kg) divided by height^2^ (m^2^). The classified physical activity questionnaire was used for the daily physical activity measurement based on metabolic equivalent to task (MET). The validity of the questionnaire was confirmed in Iran [51].

After 12-h fasting, blood samples were collected. Serum lipid levels (TC, LDL-C, HDL-C, and TG) were measured by enzymatic method (using kits from Pars Azmoon Co., Iran) [52]. The level of ghrelin and leptin were measured by the ELISA method (Bioassay Technology Co, China, and Germany) [53]. TAC measurement evaluating the overall antioxidants capacity in the body was measured by spectrophotometry and SOD levels which showes enzymatic antioxidant activity [54] was assessed using colorimetry methods (Cayman Chemical Company, USA) based on the conversion of xanthine oxidase to xanthine and O2 into uric acid and hydrogen peroxide to produce superoxide ions. Then SOD from the analyzed serum samples decomposes the superoxide ions in the reaction mixture. The results were measured spectrophotometrically and expressed as U/mL.

IL-18, PTX3, and PGF2α showing a pro-inflammatory status in the body were measured using the ELISA method (Shanghai Crystal Day Biotech Co., Ltd). The sensitivity of IL-18 and PTX3 ELISA kits was 28 ng/ l and 0.05 ng/ml, respectively. Moreover, Serum ghrelin and leptin levels were measured by ELISA method (Bioassay Technology Co, China and Mediagnost, Germany, respectively).

### Assessment of the atherogenic index of plasma (AIP) and the lipid ratio

The atherogenic index of plasma (AIP) was calculated using the logarithmic ratio of (TG to HDL-C). Furthermore, the lipid ratio was computed as follows: Castelli’s Risk Index (CRI – I) = TC/ HDL – C, CRI - II = LDL – C/HDL – C, atherogenic coefficient (AC) = (TC - HDL – C)/ HDL – C [55].

### Genotyping

Genomic DNA was isolated from whole blood by the salting-out protocol [56]. *Polymerase chain reaction* (*PCR)* was used for *genotyping* the ApoA2–265 T > C, performed by 8% polyacrylamide gel electrophoresis. Zip Nucleic Acids (ZNA) probes was used for increasing its stability and melting temperature and genotyping the 265 T > C changes of Apolipoprotein A2 gene [10, 57]. The *promoter region* of the *ApoA2 gene* containing the polymorphism *has been amplified by two pairs of primers,* upstream primer 5′CAT GGG TTG ATA TGT CAG AGC-3′ and downstream primer 5′ TCA GGT GAC AGG GAC TAT GG 3′.

### Statistical analysis

**Fig. 1:**
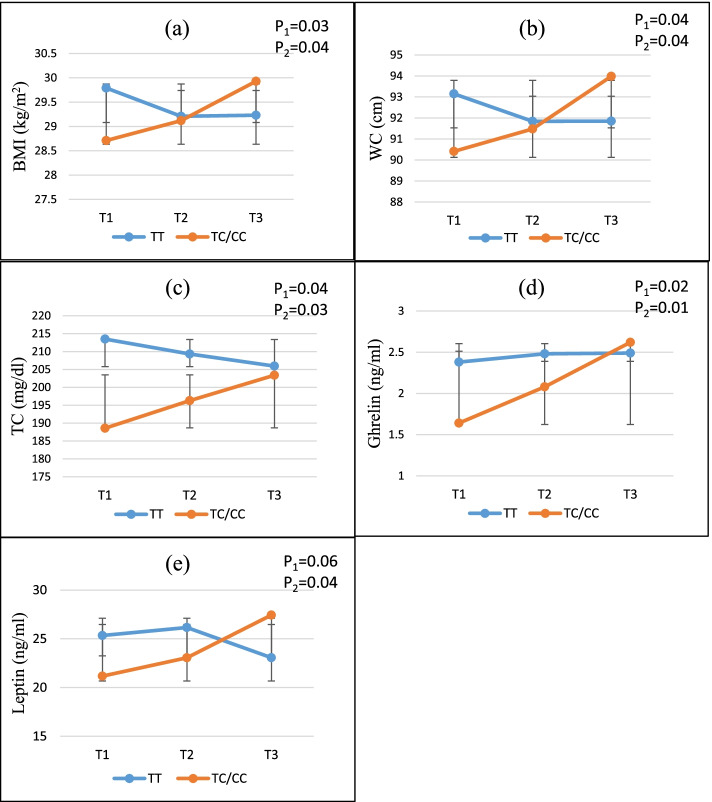
Interaction effect between NEAP (mEq/d) and APOA2–256 (C > T) on **a** BMI, **b** WC, **c** TC, **d** Ghrelin, **e** Leptin. P _1_ = *P* value with unadjusted (crude) model, P _2_ = *P* value with adjustments for potential confounding factors including (age, physical activity, sex, smoking, alcohol, energy intake, alcohol, lipid and glucose-lowering medications). The lines indicate mean ± Error bar (SD). BMI: body mass index, WC: waist circumstance, TC: total cholesterol

The Kolmogorov–Smirnov test was done to assess the normality of the data. NEAP and PRAL scores were categorized into three tertiles based on the distribution of individuals. ApoA2–265 T > C polymorphism *genotypes* were considered as *T*-*allele* carriers (*TT*/*TC*) compared to the CC genotype. The Independent T-test was used for comparison of the clinical characteristics according to ApoA2 polymorphism (Table 1). The characteristics across the quartiles of NEAP and PRAL were compared using the ANOVA (Table 2). We analyzed the interaction between NEAP/ PRAL and ApoA2–265 T > C on BMI, WC, TC, Ghrelin, and Leptin using *generalized linear models (GLM) in both* crude and adjusted models. The models were adjusted for age, physical activity, sex, smoking, alcohol, energy intake, alcohol, lipid, and glucose-lowering medications (Fig. 1). Statistical analyses were done using SPSS 16.0 software (SPSS).

**Table 1:** Comparison of the clinical characteristics according to APOA2 polymorphism

	APOA2–265 T > C		***P*** value*
N	TT = 289	(TC + CC) = 438	
**Age (year)**	53.78 ± 6.76	54.26 ± 6.49	0.33
**Alcohol (Yes)**	8 (33.3%)	16 (66.7%)	0.36
**Smoking (Yes)**	53 (39.3%)	82 (60.7%)	0.24
**BMI (kg/m2)**	29.31 ± 4.65	29.39 ± 4.78	0.82
**WC (cm)**	92.19 ± 10.36	92.32 ± 10.72	0.86
**Physical activity (MET min/week)**	37.85 ± 5.54	37.74 ± 5.42	0.79
**Total energy intake (kcal/day)**	2519.08 ± 844.92	2526.81 ± 938.86	0.91
**Protein (gr/day)**	90.21 ± 34.16	90.33 ± 36.45	0.96
**Carbohydrate (gr/day)**	339.53 ± 138.03	345.15 ± 145.92	0.59
**Total fat (gr/day)**	103.02 ± 46.72	103.76 ± 48.45	0.83
**Phosphor (mg/day)**	1677.47 ± 620.64	1683.84 ± 605.14	0.88
**Magnesium (mg/day)**	511.84 ± 227.72	508.27 ± 223.94	0.82
**Potassium (mg/day)**	4278.43 ± 1619.34	4343.19 ± 1732.50	0.6
**Calcium (mg/day)**	1153.31 ± 424.16	1168.80 ± 434.83	0.62
**EER.(Men)**	2600.70 ± 282.01	2572.46 ± 275.94	0.41
**EER.(Women)**	2020.54 ± 181.43	2022.1028 ± 183.50	0.93
**NEAP (mEq/day)**	−9.01 ± 0.26	−9.03 ± 0.24	0.26
**PRAL (mEq/day)**	−10.89 ± 21.37	−11.43 ± 22.54	0.74
**TC (mg/dl)**	203.45 ± 70.5	196.04 ± 72.29	0.21
**HDL-C (mg/dl)**	52.67 ± 11.02	53.77 ± 13.72	0.52
**LDL-C (mg/dl)**	111.6 ± 34.64	109.61 ± 76.36	0.70
**TG (mg/dl)**	184.89 ± 104.70	185.43 ± 109.28	< 0.001*
**Ghrelin (ng/ml)**	2.16 ± 0.83	2.44 ± 1.48	0.02*
**Leptin (ng/ml)**	24.51 ± 14.04	25.18 ± 14.71	0.73
**hs-CRP (mg/l)**	1.82 ± 1.23	2.48 ± 1.58	0.006*
**TAC (g/dl)**	2.51 ± 0.56	2.45 ± 0.56	0.53
**SOD (U/ml)**	0.14 ± 0.04	0.14 ± 0.04	0.29
**IL-18 (pg /ml)**	247.68 ± 33.83	249.36 ± 29.16	0.73
**PGF2α (pg/ml)**	71.60 ± 5.92	73 ± 6.19	0.16
**PTX 3 (ng/ml)**	2.81 ± 0.45	2.52 ± 0.46	< 0.001*
**AIP**	0.49 ± 0.24	0.48 ± 0.24	0.87
**AC**	3 ± 1.6	2.85 ± 1.77	0.23
**CRI-II**	2.08 ± 0.65	2.07 ± 0.67	0.93
**CR-I**	4 ± 1.6	3.85 ± 1.77	0.23

Values are means ± SD Independent T. test (*P*value*). *BMI* Body mass index, *WC* Waist circumference, *EER* Estimated Energy Requirement, *TG* Triglyceride, *TC* Total cholesterol, *hs-CRP* High sensivity c-reactive protein, *TAC* Total antioxidant capacity, *SOD* Superoxide Dismutase, *IL18* Interleukin 18, *PGF2α* Prostaglandin F2α. Atherogenic Index of Plasma (AIP) = log (TG/HDL-C), Atherogenic Coefficient (AC) = (TC-HDL-C)/HDL-C, Castelli’s Risk Index II (CRI-II) = (LDL/HDL), Castelli’s Risk Index I (CRI-I) = (TC/HDL-C)

**Table 2:** The association between metabolic markers with NEAP and PRAL in T2DM patients

	Tertile of NEAP				Tertile of PRAL			
	T1	T2	T3	***P***^***a***^	T1	T2	T3	***P***^***a***^
**N**	**242**	**242**	**242**		**242**	**242**	**242**	
**Age (year)**	54.38 ± 6.29	53.77 ± 6.56	54.09 ± 6.89	0.59	54.45 ± 5.93	53.51 ± 6.97	54.26 ± 6.84	0.25
**BMI (kg/m2)**	29.39 ± 5.01	29.13 ± 4.44	29.53 ± 4.74	0.64	29.29 ± 4.67	29.32 ± 4.97	29.47 ± 4.55	0.90
**WC (cm)**	92.05 ± 11.27	91.60 ± 9.88	93.13 ± 10.54	0.26	91.47 ± 10.57	92.05 ± 10.80	93.29 ± 10.32	0.15
**Physical activity (MET min/week)**	38.09 ± 5.08	38.09 ± 5.58	37.14 ± 5.70	0.08	38.81 ± 5.7	37.27 ± 4.91	37.28 ± 5.62	**0.002**
**Energy intake (kcal/day)**	2403.72 ± 823.39	2457.27 ± 736.39	2710.80 ± 1084.58	**< 0.001**	2622.06 ± 876.52	2271.40 ± 693.04	2679.12 ± 1051.77	**< 0.001**
**EER.(Men)**	2548.14 ± 276.67	2553.32 ± 258.79	2635.04 ± 290.23	**0.04**	2553.65 ± 279.22	2552.15 ± 253.93	2631.33 ± 291.47	0.06
**EER.(Women)**	2010.66 ± 189.44	2016.83 ± 161.13	2038.14 ± 193.95	0.41	2008.6 ± 175.19	2018.46 ± 176.05	2040.61 ± 197.73	0.32
**HDL-C (mg/dl)**	54.76 ± 13.44	52.35 ± 12.59	52.68 ± 11.73	0.07	55.45 ± 13.81	51.59 ± 11.86	52.76 ± 11.86	**0.003**
**LDL-C (mg/dl)**	110.18 ± 37.86	106.72 ± 35.41	105.96 ± 32.16	0.37	111.14 ± 39.10	105.39 ± 33.97	106.89 ± 32.88	0.18
**TC (mg/dl)**	195.70 ± 68.43	203.88 ± 75.79	197.76 ± 77.63	0.45	197.38 ± 70.67	200.93 ± 73.10	199.27 ± 78.34	0.87
**TG (mg/dl)**	184.54 ± 107.98	185.52 ± 106.84	185.97 ± 108.05	0.98	190.78 ± 109.63	177.41 ± 100.91	187.39 ± 111.41	0.37
**Leptin (ng/ml)**	25.76 ± 13.61	25.48 ± 14.75	23.16 ± 15.26	0.48	25.54 ± 13.52	25.38 ± 15.49	23.56 ± 14.55	0.65
**Ghrelin (ng/ml)**	2.32 ± 1.02	2.32 ± 1.44	2.44 ± 1.47	0.83	2.16 ± 0.95	2.51 ± 1.45	2.42 ± 1.54	0.18
**hs-CRP (mg/L)**	2.31 ± 1.41	2.18 ± 1.59	2.24 ± 1.52	0.09	2.20 ± 1.41	2.28 ± 1.57	2.26 ± 1.52	0.96
**PTX3(ng/ml)**	2.6 ± 0.48	2.66 ± 0.46	2.60 ± 0.49	0.72	2.56 ± 0.51	2.67 ± 0.44	2.61 ± 0.47	0.47
**IL18(pg/ml)**	250.74 ± 37.67	245.75 ± 26.09	250.69 ± 27.55	0.61	248.61 ± 37.47	247.30 ± 27.18	250.55 ± 28.07	0.84
**TAC(g/dl)**	2.55 ± 0.53	2.45 ± 0.58	2.44 ± 0.56	0.54	2.61 ± 0.59	2.41 ± 0.52	2.42 ± 0.56	0.13
**SOD(U/ml)**	0.14 ± 0.03	0.14 ± 0.04	0.15 ± 0.04	0.40	0.14 ± 0.03	0.13 ± 0.04	0.15 ± 0.04	0.34
**PGF2α(pg/ml)**	72.83 ± 6.05	73.25 ± 5.76	71.39 ± 6.56	0.25	72.80 ± 6.02	73.21 ± 5.85	71.46 ± 6.48	0.28
**AIP**	0.48 ± 0.24	0.49 ± 0.25	0.49 ± 0.25	0.74	0.49 ± 0.23	0.48 ± 0.24	0.49 ± 0.26	0.98
**AC**	2.75 ± 1.59	3.07 ± 1.82	2.91 ± 1.70	0.13	2.75 ± 1.66	3.06 ± 1.74	2.93 ± 1.71	0.14
**CRI.II**	2.06 ± 0.68	2.09 ± 0.67	2.06 ± 0.64	0.88	2.05 ± 0.7	2.09 ± 0.66	2.08 ± 0.64	0.85
**CRI**	3.75 ± 1.59	4.07 ± 1.82	3.91 ± 1.70	0.13	3.75 ± 1.66	4.06 ± 1.74	3.93 ± 1.71	0.14

Data are presented as mean ± standard deviation (SD). *Abbreviation*: *PRAL* Potential renal acid load, *NEAP* Net endogenous acid production, *BMI* Body mass index, *WC* Waist circumference, *HDL-C* High density lipoprotein cholesterol, *LDL-C* Low density lipoprotein cholesterol, *CH* Cholesterol, *TG* Triglyceride, *hs-CRP* High sensivity C-reactive protein, *PTX3* Pentraxin 3, *IL18* Interleukin 18, *TAC* Total antioxidant capacity, *SOD* Superoxide dismutase, *PGF2α* ProstaglandinF2α. Atherogenic Index of Plasma (AIP) = log (TG/HDL-C), Atherogenic Coefficient (AC) = (TC-HDL-C)/HDL-C, Castelli’s Risk Index II (CRI-II) = (LDL/HDL), Castelli’s Risk Index I (CRI-I) = (TC/HDL-C) ^a^ Obtained from ANOVA

## Results

### Associations between cardio-metabolic markers and ApoA2–265 T > C polymorphism

A total of 727 patients with T2DM participated in this study. The frequency overall prevalence of rs5082 genotypes was 63.82 and 36.17% for T-allele and C-allele respectively. The genotype distributions were within HWE (*P*-value > 0.05). Details of the biochemical variables between rs5082 genotypes are presented in Table 1. TG, Ghrelin, and hs-CRP concentrations were significantly higher among C allele carriers than TT homozygotes. However, TC/CC genotypes have lower PTX3 than TT homozygotes. Additionally, there were no significant differences for other variables, according to ApoA2 genotypes.

### Association between cardio-metabolic markers and NEAP and PRAL

The basic information of diabetic patients between the NEAP and PRAL groups is presented in Table 2. All patients were divided into three groups, based on their NEAP and PRAL scores. Patients in the third tertiles of NEAP and PRAL had higher total energy intake (*p* < 0.001). Regarding PRAL, patients in the last tertile were more likely to have lower activity (*P* = 0.002) and more adherence to PRAL reduced HDL-C (*p* = 0.003). There was a significant difference in EER across the NEAP tertiles. Men in the last tertiles of NEAP had more EER, compared to the first tertiles of NEAP. There were no significant associations found regarding other basic characteristics and biochemical parameters between the NEAP and PRAL groups.

### Interaction between NEAP and PRAL with ApoA2–265 T > C on cardio-metabolic markers

**Fig. 2:**
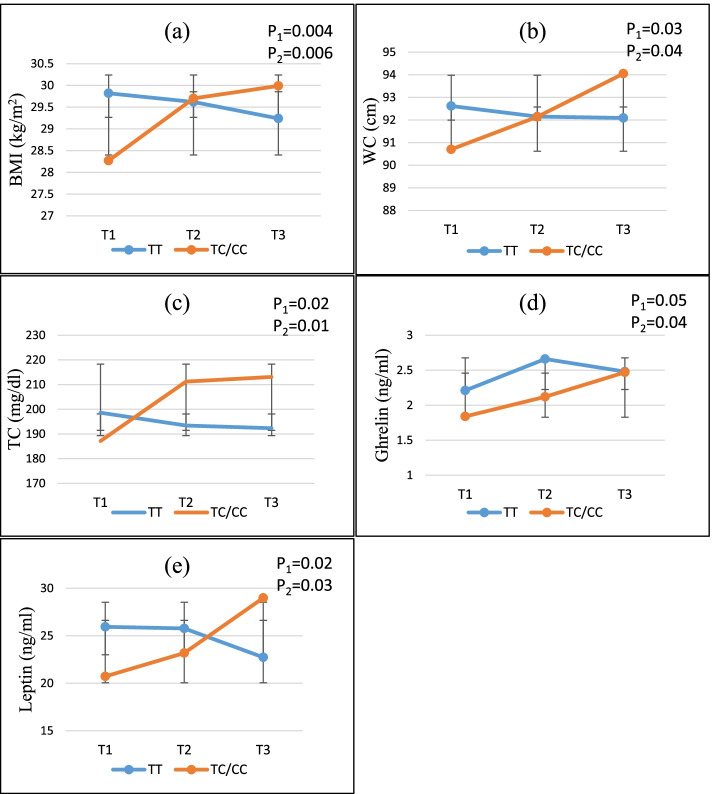
Interaction effect between PRAL (mEq/d) and APOA2–256 (C > T) on **a** BMI, **b** WC, **c** TC, **d** Ghrelin, **e** Leptin. P _1_ = *P* value with unadjusted (crude) model, P _2_ = *P* value with adjustments for potential confounding factors including (age, physical activity, sex, smoking, alcohol, energy intake, alcohol, lipid and glucose-lowering medications). The lines indicate mean ± Error bar (SD). BMI: body mass index, WC: waist circumstance, TC: total cholesterol

The interaction between ApoA2–265 T > C polymorphism and tertiles of NEAP and PRAL scores on cardio-metabolic marker was shown in Figs. 1 and 2. Significant interactions were observed between NEAP and PRAL score and rs5082 SNP in terms of BMI, WC, TC, leptin and ghrelin in both crude and adjusted models. This study revealed that those with the TC/CC genotype had higher BMI (P_1_-interaction =0.03, P_2_-interaction =0.04) (Fig. 1a), WC (P_1_-interaction =0.04, P_2_-interaction =0.04) (Fig. 1b), TC (P_1_-interaction =0.04, P_2_-interaction =0.03) (Fig. 1c), ghrelin (P_1_-interaction =0.02, P_2_-interaction =0.01) (Fig. 1d) and leptin (P_1_-interaction =0.06, P_2_-interaction =0.04) (Fig. 1e) when they consumed diets higher on the NEAP index. Moreover, the highest tertiles of the PRAL, compared to the lowest, showed increased in BMI (P_1_-interaction =0.004, P_2_-interaction =0.006) (Fig. 2a), WC (P_1_-interaction =0.03, P_2_-interaction =0.04) (Fig. 2b), TC (P_1_-interaction =0.02, P_2_-interaction =0.01) (Fig. 2c), ghrelin (P_1_-interaction =0.05, P_2_-interaction =0.04) (Fig. 2d), and leptin (P_1_-interaction =0.02, P_2_-interaction =0.03) (Fig. 2e), for TC/CC genotypes compare those with TT homozygotes. However, In − 265 T > C polymorphism, no significant difference was observed in other metabolic markers between the different groups of DAL intake.

## Discussion

For all we know, this study is the first attempt to investigate the interplay effect of NEAP, PRAL, and ApoA2–265 T > C on cardio-metabolic markers in individuals with T2DM. Based on findings, C carriers had significantly high TG, ghrelin, and hs-CRP, and lower PTX3 versus TT homozygous. Moreover, those with less DAL had better HDL-C levels. The results of the current study demonstrated that ApoA2–265 T > C polymorphism may alter the effect of the DAL on BMI, WC, TC, ghrelin, and leptin. In particular, C carriers had the highest mean of BMI, WC, TC, ghrelin, and leptin when placed in top tertiles of NEAP and PRAL. The locus on the 1q21-q23 chromosome relates to ApoA2 which substitution of T to C at 265 bp before the ApoA2 gene transcription forms ApoA2–265 T > C polymorphism. The -265 T > C is mostly notified between the variety of SNPs of the ApoA2 gene, which is associated with reduced serum ApoA2 levels [58, 59]. It has been stated that this reduction might be the leading cause of elevated hs-CRP in C carriers [60–62]. Consistent with our findings, Basiri et al. reported a highly significant level of ghrelin in CC homozygous [63]. Ghrelin is known as an orexigenic and appetite hormone, thereby, a high serum level of ghrelin was expected in C carriers against TT homozygous. The other finding of this study was the inverse association between HDL-C concentration and tertiles of DAL indexes. The findings of various studies are a point of contention. Along with Kucharska et al., different studies suggested the same trend of HDL-C by increasing NEAP and PRAL scores [40, 41] which were in line with the result of the present study, however, some other conflicting results were presented in previous studies [42, 64–66] which further investigations required to give insight into the relationship between HDL-C concentration and DAL. The interplay effect of the genotypes with DAL has not been assessed so far, nevertheless, the interaction effect of the polymorphism with dietary intake on the aforementioned markers was checked in several studies. Besides, multiple studies considered the association of those markers with DAL. Based on findings of previous studies, CC homozygous with a greater intake of n-3 PUFA tend to have a decreased level of IL-18 and hs-CRP whereas higher intake of saturated fatty acids was related to increased BMI, LDL-C, and LDL-C to HDL-C ratio [44, 45, 67].

The findings on the interplay effect of ApoA2–265 T > C and DAL on obesity markers were in line with various studies. The study conducted by Murakami et al. obtained a marginally significant relationship of DAL with WC [68]. Higher PRAL and NEAP scores linked with increased BMI through Farhangi et al. findings [42]. Moreover, a greater odds of obesity was reported through median and quartiles of NEAP [43, 65, 69]. This might be explained by the association of a western dietary pattern containing a high amount of refined grains, red meats, and eggs with obesity [70, 71]. Additionally, metabolic acidosis promotes muscle mass loss by downregulating protein synthesis and developing proteolysis in terms of either ubiquitin-proteasome system or IGF-1 signaling alterations [72]. Furthermore, C allele carriers had a high consumption of red meat products in this study. On the one hand, the relationship between red meat consumption and DAL has been revealed in different studies [35, 73, 74]. On the other hand, it has been remarked that red meat consumption exacerbates a pro-inflammatory state and is consequently associated with a high BMI and WC [75–78]. Adding to this, C carriers in the current study consumed more added sugars than others. Several studies suggested the association between added sugars and elevated inflammatory markers e.g. hs-CRP [79, 80]. Notably, elevated pro-inflammatory markers were also observed in obese adults in multiple studies [81]. On top of that, various studies have reported an augmented risk of obesity in CC homozygotes hardly by consumption of high amounts of saturated fatty acids and they are more vulnerable to consuming more food, particularly more high-fat and high-protein foods that manifest a western dietary pattern [15, 21, 44, 82, 83]. Studies proposed that these subjects had up-regulated methylation at cg04436964, close to ApoA2 gene, versus T carriers which linked with down-regulated ApoA2 expression [84] that might be a justification for obesity trait by gene-diet interaction.

The current findings also demonstrated CC homozygotes had an increased level of TC when placed in the top tertile of NEAP/PRAL. It was noted that subjects with a low score of DAL or adherence to a DASH/plant-based diet tend to have dropped TC levels in several studies [68, 85–87]. It is noteworthy to point out the high correspondence between low DAL and DASH/plant-based diets [42, 88, 89]. Reducing SFA intake by decreasing meat-derived protein consumption – with the potential of intensifying inflammatory responses- might clarify the decreased level of TC through low adherence to DAL. Moreover, a high oxidative stress level, which could be a determiner of inflammation, was correlated positively with high TC [90–92].

Regarding the hormones, the plasma levels of leptin and ghrelin were affected by the interplay effect of the polymorphism and DAL. CC homozygotes had the highest level of leptin and ghrelin by adherence to DAL. Consistent with the findings, the plasma level of leptin was lower in those who adopted with vegetarian dietary pattern compared with non-vegetarians [93, 94]. As mentioned before, red meat consumption promotes diet acidity and inflammatory responses. A linkage between high PRAL or NEAP scores with an increased hs-CRP was reported previously [95]. This association could be explained by metabolic acidosis which might contribute to the initiation of the inflammatory responses by provoking tissue damage [96]. Hs-CRP is also positively linked with leptin. Leptin is secreted by the adipose tissue and adipocytes are one of the main sources of hs-CRP secretion which leptin could intensify the production of hs-CRP as well [97–99]. Furthermore, obesity as a result of greater energy intake is associated with an elevated level of leptin. With respect to ghrelin, it has been introduced as an effective anti-inflammatory hormone by numerous investigations [100]. Accordingly, ghrelin level increased expectedly in C carriers due to the elevation of hs-CRP to downregulate the inflammation.

Despite the novelty of the findings, the current study had some limitations. Primarily, this cross-sectional study did not measure the ApoA2 serum concentration and insulin. Furthermore, utilizing the FFQ for dietary intake evaluation has potentially recall bias along with over- or under-report of participants. Conclusively, a dietary pattern with a high acid load interacts with ApoA2 genotypes and could significantly impact BMI, WC, TC, leptin, and ghrelin. These findings warrant confirmation in high-quality interventional studies.

## Conclusion

Based on the findings of this study, the ApoA2 polymorphism may be associated with CVD risk factors in T2DM patients with high dietary acid indices, such as NEAP and PRAL. This finding suggests that the ApoA2–265 T > C (TC + CC) allele may exacerbate the CVD risk posed by elevated NEAP and PRAL levels. This is important for clinical diagnosis and gene-based treatment.

## Data Availability

The data are not publicly available due to containing private information of participants. Data are however available from the authors upon reasonable request and with permission of Fariba Koohdani.

## References

[1] Esteghamati A, Larijani B, Aghajani MH, Ghaemi F, Kermanchi J, Shahrami A, Saadat M, Esfahani EN, Ganji M, Noshad S (2017). Diabetes in Iran: prospective analysis from first nationwide diabetes report of National Program for prevention and control of diabetes (NPPCD-2016). Sci Rep.

[2] Naeini Z, Toupchian O, Vatannejad A, Sotoudeh G, Teimouri M, Ghorbani M, Nasli-Esfahani E, Koohdani F (2020). Effects of DHA-enriched fish oil on gene expression levels of p53 and NF-κB and PPAR-γ activity in PBMCs of patients with T2DM: a randomized, double-blind, clinical trial. Nutr Metab Cardiovasc Dis.

[3] Leon BM, Maddox TM (2015). Diabetes and cardiovascular disease: epidemiology, biological mechanisms, treatment recommendations and future research. World J Diabetes.

[4] Giacco F, Brownlee M (2010). Oxidative stress and diabetic complications. Circ Res.

[5] Tiwari BK, Pandey KB, Abidi AB, Rizvi SI. Markers of Oxidative Stress during Diabetes Mellitus. J Biomark. 2013;2013:378790. 10.1155/2013/378790. 10.1155/2013/378790 PMC4437365 26317014

[6] Matough FA, Budin SB, Hamid ZA, Alwahaibi N, Mohamed J (2012). The role of oxidative stress and antioxidants in diabetic complications. Sultan Qaboos Univ Med J.

[7] Jafari Azad B, Yaseri M, Daneshzad E, Koohdani F. Interaction between Apo A-II -265T>C polymorphism and dietary total antioxidant capacity on some anthropometric indices and serum lipid profile in patients with type 2 diabetes mellitus. J Nutr Sci. 2021;10:e9. 10.1017/jns.2020.61. 10.1017/jns.2020.61 PMC8057501 33889392

[8] Murea M, Ma L, Freedman BI (2012). Genetic and environmental factors associated with type 2 diabetes and diabetic vascular complications. Rev Diabet Stud.

[9] Kido Y (2017). Gene–environment interaction in type 2 diabetes. Diabetol Int.

[10] Basiri MG, Sotoudeh G, Alvandi E, Djalali M, Eshraghian MR, Noorshahi N, Koohdani F (2015). APOA2− 256T> C polymorphism interacts with saturated fatty acids intake to affect anthropometric and hormonal variables in type 2 diabetic patients. Genes Nutr.

[11] Noorshahi N, Sotoudeh G, Djalali M, Eshraghian MR, Keramatipour M, Basiri MG, Doostan F, Koohdani F (2016). APOA II genotypes frequency and their interaction with saturated fatty acids consumption on lipid profile of patients with type 2 diabetes. Clin Nutr.

[12] Zaki ME, Amr KS, Abdel-Hamid M (2014). Evaluating the association of APOA2 polymorphism with insulin resistance in adolescents. Meta Gene.

[13] Duesing K, Charpentier G, Marre M, Tichet J, Hercberg S, Balkau B, Froguel P, Gibson F (2009). Evaluating the association of common APOA2 variants with type 2 diabetes. BMC Med Genet.

[14] Sotos-Prieto M, Peñalvo JL (2013). Genetic variation of apolipoproteins, diet and other environmental interactions; an updated review. Nutr Hosp.

[15] Corella D, Arnett DK, Tsai MY, Kabagambe EK, Peacock JM, Hixson JE, Straka RJ, Province M, Lai C-Q, Parnell LD (2007). The− 256T> C polymorphism in the apolipoprotein A-II gene promoter is associated with body mass index and food intake in the genetics of lipid lowering drugs and diet network study. Clin Chem.

[16] Zaki ME, Amr KS, Abdel-Hamid M (2013). APOA2 polymorphism in relation to obesity and lipid metabolism. Cholesterol.

[17] Julve-Gil J, Ruiz-Pérez E, Casaroli-Marano RP, Marzal-Casacuberta À, Escolà-Gil JC, González-Sastre F, Blanco-Vaca F (1999). Free cholesterol deposition in the cornea of human apolipoprotein A-II transgenic mice with functional lecithin: cholesterol acyltransferase deficiency. Metabolism.

[18] Julve J, Marzal-Casacuberta À, Ordóñez-Llanos J, González-Sastre F, Blanco-Vaca F (2001). ApoA-II expression in CETP transgenic mice increases VLDL production and impairs VLDL clearance. J Lipid Res.

[19] Boisfer E, Lambert G, Atger V, Tran NQ, Pastier D, Benetollo C, Trottier J-F, Beaucamps I, Antonucci M, Laplaud M (1999). Overexpression of human apolipoprotein A-II in mice induces hypertriglyceridemia due to defective very low density lipoprotein hydrolysis. J Biol Chem.

[20] Basiri MG, Sotoudeh G, Alvandi E, Djalali M, Eshraghian MR, Noorshahi N, Koohdani F (2015). APOA2− 256T> C polymorphism interacts with saturated fatty acids intake to affect anthropometric and hormonal variables in type 2 diabetic patients. Genes Nutr.

[21] Smith C, Ordovas J, Sanchez-Moreno C, Lee Y, Garaulet M (2012). Apolipoprotein A-II polymorphism: relationships to behavioural and hormonal mediators of obesity. Int J Obes.

[22] Klok MD, Jakobsdottir S, Drent M (2007). The role of leptin and ghrelin in the regulation of food intake and body weight in humans: a review. Obes Rev.

[23] Eckstein P (2011). The role of ghrelin and leptin in obesity: is exogenous administration of these hormones a possible drug therapy?. Sci J Lander Coll Arts Sci.

[24] Adamska-Patruno E, Ostrowska L, Goscik J, Fiedorczuk J, Moroz M, Kretowski A, Gorska M (2019). The differences in postprandial serum concentrations of peptides that regulate satiety/hunger and metabolism after various meal intake, in men with normal vs. excessive BMI. Nutrients.

[25] Gargari BP, Houjeghani S, Farzadi L, Houjeghani S, Safaeiyan A (2015). Relationship between serum leptin, ghrelin and dietary macronutrients in women with polycystic ovary syndrome. Int J Fertil Steril.

[26] Kong A, Neuhouser ML, Xiao L, Ulrich CM, McTiernan A, Foster-Schubert KE (2009). Higher habitual intake of dietary fat and carbohydrates are associated with lower leptin and higher ghrelin concentrations in overweight and obese postmenopausal women with elevated insulin levels. Nutr Res.

[27] Polak AM, Krentowska A, Łebkowska A, Buczyńska A, Adamski M, Adamska-Patruno E, Fiedorczuk J, Krętowski AJ, Kowalska I, Adamska A (2020). The association of serum levels of leptin and ghrelin with the dietary fat content in non-obese women with polycystic ovary syndrome. Nutrients.

[28] Domínguez-Reyes T, Astudillo-López CC, Salgado-Goytia L, Muñoz-Valle JF, Salgado-Bernabé AB, Guzmán-Guzmán IP, Castro-Alarcón N, Moreno-Godínez ME, Parra-Rojas I (2015). Interaction of dietary fat intake with APOA2, APOA5 and LEPR polymorphisms and its relationship with obesity and dyslipidemia in young subjects. Lipids Health Dis.

[29] Jafari Azad B, Yaseri M, Daneshzad E, Koohdani F (2021). Interaction between Apo A-II -265T>C polymorphism and dietary total antioxidant capacity on some anthropometric indices and serum lipid profile in patients with type 2 diabetes mellitus. J Nutr Sci.

[30] Emamat H, Tangestani H, Bahadoran Z, Khalili-Moghadam S, Mirmiran P (2019). The associations of dietary acid load with insulin resistance and type 2 diabetes: a systematic review of existing human studies. Recent Pat Food Nutr Agric.

[31] Osuna-Padilla I, Leal-Escobar G, Garza-García C, Rodríguez-Castellanos F (2019). Dietary acid load: mechanisms and evidence of its health repercussions. Nefrología (Engl Ed).

[32] Hietavala E, Stout J, Hulmi J, Suominen H, Pitkänen H, Puurtinen R, Selänne H, Kainulainen H, Mero A (2015). Effect of diet composition on acid–base balance in adolescents, young adults and elderly at rest and during exercise. Eur J Clin Nutr.

[33] Mirzababaei A, Shiraseb F, Setayesh L, Tavakoli A, Daneshzad E, Abaj F, Clark CCT, Mirzaei K (2022). The association of dietary acid load with resting metabolic rate and metabolic components in overweight and obese women: a cross sectional study. Clin Nutr ESPEN.

[34] Abshirini M, Bagheri F, Mahaki B, Siassi F, Koohdani F, Safabakhsh M, Sotoudeh G (2019). The dietary acid load is higher in subjects with prediabetes who are at greater risk of diabetes: a case–control study. Diabetol Metab Syndr.

[35] Kiefte-de Jong JC, Li Y, Chen M, Curhan GC, Mattei J, Malik VS, Forman JP, Franco OH, Hu FB (2017). Diet-dependent acid load and type 2 diabetes: pooled results from three prospective cohort studies. Diabetologia.

[36] Tsalamandris S, Antonopoulos AS, Oikonomou E, Papamikroulis G-A, Vogiatzi G, Papaioannou S, Deftereos S, Tousoulis D (2019). The role of inflammation in diabetes: current concepts and future perspectives. Eur Cardiol Rev.

[37] Zlibut A, Bocsan IC, Agoston-Coldea L (2019). Pentraxin-3 and endothelial dysfunction. Adv Clin Chem.

[38] Moussavi Javardi MS, Madani Z, Movahedi A, Karandish M, Abbasi B (2020). The correlation between dietary fat quality indices and lipid profile with Atherogenic index of plasma in obese and non-obese volunteers: a cross-sectional descriptive-analytic case-control study. Lipids Health Dis.

[39] Lee H, Woo J, Chen Z, Leung S, Peng X (2000). Serum fatty acid, lipid profile and dietary intake of Hong Kong Chinese omnivores and vegetarians. Eur J Clin Nutr.

[40] Kucharska AM, Szostak-Węgierek DE, Waśkiewicz A, Piotrowski W, Stepaniak U, Pająk A, Kozakiewicz K, Tykarski A, Rutkowski M, Bielecki WJ (2018). Dietary acid load and cardiometabolic risk in the polish adult population. Adv Clin Exp Med.

[41] Moghadam SK, Bahadoran Z, Mirmiran P, Tohidi M, Azizi F (2016). Association between dietary acid load and insulin resistance: Tehran lipid and glucose study. Prev Nutr Food Sci.

[42] Abbasalizad Farhangi M, Nikniaz L, Nikniaz Z (2019). Higher dietary acid load potentially increases serum triglyceride and obesity prevalence in adults: an updated systematic review and meta-analysis. PLoS One.

[43] Arisawa K, Katsuura-Kamano S, Uemura H, Van Tien N, Hishida A, Tamura T, Kubo Y, Tsukamoto M, Tanaka K, Hara M (2020). Association of dietary acid load with the prevalence of metabolic syndrome among participants in baseline survey of the Japan multi-institutional collaborative cohort study. Nutrients.

[44] Corella D, Peloso G, Arnett DK, Demissie S, Cupples LA, Tucker K, Lai CQ, Parnell LD, Coltell O, Lee YC (2009). APOA2, dietary fat, and body mass index: replication of a gene-diet interaction in 3 independent populations. Arch Intern Med.

[45] Keramat L, Sadrzadeh-Yeganeh H, Sotoudeh G, Zamani E, Eshraghian M, Mansoori A, Koohdani F (2017). Apolipoprotein A2–265 T>C polymorphism interacts with dietary fatty acids intake to modulate inflammation in type 2 diabetes mellitus patients. Nutrition.

[46] Esmaillzadeh A, Mirmiran P, Azizi F (2005). Whole-grain consumption and the metabolic syndrome: a favorable association in Tehranian adults. Eur J Clin Nutr.

[47] Frassetto LA, Todd KM, Morris RC, Sebastian A (1998). Estimation of net endogenous noncarbonic acid production in humans from diet potassium and protein contents. Am J Clin Nutr.

[48] Remer T, Dimitriou T, Manz F (2003). Dietary potential renal acid load and renal net acid excretion in healthy, free-living children and adolescents. Am J Clin Nutr.

[49] Willett WC, Howe GR, Kushi LH (1997). Adjustment for total energy intake in epidemiologic studies. Am J Clin Nutr.

[50] Organization WH (2011). Waist circumference and waist-hip ratio: report of a WHO expert consultation, Geneva, 8-11 December 2008.

[51] Kelishadi R, Rabiee K, Khosravi A, Famori F, Sadeghi M, Roohafza H (2001). Association of physical activity pattern of adolecencein Isfahan. Shahre ord Univ Med Sci.

[52] Abaj F, Rafiee M, Koohdani F (2021). Interaction between dietary total antioxidant capacity and BDNF Val66Met polymorphism on lipid profiles and atherogenic indices among diabetic patients. Sci Rep.

[53] Abaj F, Rafiee M, Koohdani F (2021). Interaction between CETP polymorphism and dietary insulin index and load in relation to cardiovascular risk factors in diabetic adults. Sci Rep.

[54] Godeny P, Lozovoy MAB, Dichi JB, Dichi I (2010). Effect of n-3 fatty acids in glycemic and lipid profiles, oxidative stress and total antioxidant capacity in patients with the metabolic syndrome. Arq Bras Endocrinol Metabol.

[55] Olamoyegun MA, Oluyombo R, Asaolu SO (2016). Evaluation of dyslipidemia, lipid ratios, and atherogenic index as cardiovascular risk factors among semi-urban dwellers in Nigeria. Ann Afr Med.

[56] Miller S, Dykes D, Polesky H (1988). A simple salting out procedure for extracting DNA from human nucleated cells. Nucleic Acids Res.

[57] Alvandi E, Koohdani F (2014). Zip nucleic acid: a new reliable method to increase the melting temperature of real-time PCR probes. J Diabet Metab Disord.

[58] Takada D, Emi M, Ezura Y, Nobe Y, Kawamura K, Iino Y, Katayama Y, Xin Y, Wu LL, Larringa-Shum S (2002). Interaction between the LDL-receptor gene bearing a novel mutation and a variant in the apolipoprotein A-II promoter: molecular study in a 1135-member familial hypercholesterolemia kindred. J Hum Genet.

[59] van’t Hooft FM, Ruotolo G, Boquist S, de Faire U, Eggertsen G, Hamsten A (2001). Human evidence that the apolipoprotein a-II gene is implicated in visceral fat accumulation and metabolism of triglyceride-rich lipoproteins. Circulation.

[60] Birjmohun RS, Dallinga-Thie GM, Kuivenhoven JA, Stroes E, Otvos JD, Wareham NJ, Luben R, Kastelein J, Khaw K-T, Boekholdt SM (2007). Apolipoprotein A-II is inversely associated with risk of future coronary artery disease. Circulation.

[61] Onat A, Hergenç G, Ayhan E, Uğur M, Can G (2009). Impaired anti-inflammatory function of apolipoprotein A-II concentrations predicts metabolic syndrome and diabetes at 4 years follow-up in elderly Turks. Clin Chem Lab Med.

[62] Yi DW, Jeong DW, Lee SY, Son SM, Kang YH (2012). The association between apolipoprotein A-II and metabolic syndrome in Korean adults: a comparison study of apolipoprotein A-I and apolipoprotein B. Diabet Metab J.

[63] Basiri MG, Sotoudeh G, Alvandi E, Djalali M, Eshraghian MR, Noorshahi N, Koohdani F (2015). APOA2 -256T>C polymorphism interacts with saturated fatty acids intake to affect anthropometric and hormonal variables in type 2 diabetic patients. Genes Nutr.

[64] Daneshzad E, Haghighatdoost F, Azadbakht L (2019). Dietary acid load and cardiometabolic risk factors: a systematic review and meta-analysis of observational studies. Public Health Nutr.

[65] Mozaffari H, Namazi N, Larijani B, Bellissimo N, Azadbakht L (2019). Association of dietary acid load with cardiovascular risk factors and the prevalence of metabolic syndrome in Iranian women: a cross-sectional study. Nutrition.

[66] Bahadoran Z, Mirmiran P, Khosravi H, Azizi F (2015). Associations between dietary Acid-Base load and Cardiometabolic risk factors in adults: the Tehran lipid and glucose study. Endocrinol Metab (Seoul).

[67] Basiri MG, Sotoudeh G, Alvandi E, Djalali M, Eshraghian MR, Noorshahi N, Koohdani F (2015). APOA2–256T>C polymorphism interacts with saturated fatty acids intake to affect anthropometric and hormonal variables in type 2 diabetic patients. Genes Nutr.

[68] Murakami K, Sasaki S, Takahashi Y, Uenishi K (2008). Association between dietary acid-base load and cardiometabolic risk factors in young Japanese women. Br J Nutr.

[69] Fatahi S, Qorbani M, Azadbakht L (2019). Association between dietary acid load with weight status, dietray quality index (DQI), mean adequacy ratio and energy density among women. J Gorgan Univ Med Sci.

[70] McNaughton SA, Ball K, Mishra GD, Crawford DA (2008). Dietary patterns of adolescents and risk of obesity and hypertension. J Nutr.

[71] Ambrosini GL, Huang RC, Mori TA, Hands BP, O'Sullivan TA, de Klerk NH, Beilin LJ, Oddy WH (2010). Dietary patterns and markers for the metabolic syndrome in Australian adolescents. Nutr Metab Cardiovasc Dis.

[72] Bailey JL, Wang X, England BK, Price SR, Ding X, Mitch WE (1996). The acidosis of chronic renal failure activates muscle proteolysis in rats by augmenting transcription of genes encoding proteins of the ATP-dependent ubiquitin-proteasome pathway. J Clin Invest.

[73] Rebholz CM, Coresh J, Grams ME, Steffen LM, Anderson CAM, Appel LJ, Crews DC (2015). Dietary acid load and incident chronic kidney disease: results from the ARIC study. Am J Nephrol.

[74] Chen SW, Chen ZH, Liang YH, Wang P, Peng JW (2019). Elevated hypertension risk associated with higher dietary acid load: a systematic review and meta-analysis. Clin Nutr ESPEN.

[75] Mazidi M, Kengne AP, George ES, Siervo M (2021). The association of red meat intake with inflammation and circulating intermediate biomarkers of type 2 diabetes is mediated by central adiposity. Br J Nutr.

[76] Babio N, Sorlí M, Bulló M, Basora J, Ibarrola-Jurado N, Fernández-Ballart J, Martínez-González MA, Serra-Majem L, González-Pérez R, Salas-Salvadó J (2012). Association between red meat consumption and metabolic syndrome in a Mediterranean population at high cardiovascular risk: cross-sectional and 1-year follow-up assessment. Nutr Metab Cardiovasc Dis.

[77] Ley SH, Sun Q, Willett WC, Eliassen AH, Wu K, Pan A, Grodstein F, Hu FB (2014). Associations between red meat intake and biomarkers of inflammation and glucose metabolism in women. Am J Clin Nutr.

[78] Montonen J, Boeing H, Fritsche A, Schleicher E, Joost HG, Schulze MB, Steffen A, Pischon T (2013). Consumption of red meat and whole-grain bread in relation to biomarkers of obesity, inflammation, glucose metabolism and oxidative stress. Eur J Nutr.

[79] Yang Q, Zhang Z, Gregg EW, Flanders WD, Merritt R, Hu FB (2014). Added sugar intake and cardiovascular diseases mortality among US adults. JAMA Intern Med.

[80] Lin W-T, Kao Y-H, Sothern MS, Seal DW, Lee C-H, Lin H-Y, Chen T, Tseng T-S (2020). The association between sugar-sweetened beverages intake, body mass index, and inflammation in US adults. Int J Public Health.

[81] Asghar A, Sheikh N (2017). Role of immune cells in obesity induced low grade inflammation and insulin resistance. Cell Immunol.

[82] Corella D, Tai ES, Sorlí JV, Chew SK, Coltell O, Sotos-Prieto M, García-Rios A, Estruch R, Ordovas JM (2011). Association between the APOA2 promoter polymorphism and body weight in Mediterranean and Asian populations: replication of a gene–saturated fat interaction. Int J Obes.

[83] Smith CE, Tucker KL, Arnett DK, Noel SE, Corella D, Borecki IB, Feitosa MF, Aslibekyan S, Parnell LD, Lai CQ (2013). Apolipoprotein A2 polymorphism interacts with intakes of dairy foods to influence body weight in 2 U.S. populations. J Nutr.

[84] Lai C-Q, Smith CE, Parnell LD, Lee Y-C, Corella D, Hopkins P, Hidalgo BA, Aslibekyan S, Province MA, Absher D (2018). Epigenomics and metabolomics reveal the mechanism of the APOA2-saturated fat intake interaction affecting obesity. Am J Clin Nutr.

[85] Tehrani H, Haghighatdoost F, Moosavian SP, Azadbakht L, Saraf-Bank S (2018). The acidity of early pregnancy diet and risk of gestational diabetes mellitus. Clin Nutr.

[86] Perry CA, Van Guilder GP, Hossain M, Kauffman A (2021). Cardiometabolic changes in response to a calorie-restricted DASH diet in obese older adults. Front Nutr.

[87] Wright N, Wilson L, Smith M, Duncan B, McHugh P (2017). The BROAD study: a randomised controlled trial using a whole food plant-based diet in the community for obesity, ischaemic heart disease or diabetes. Nutr Diabet.

[88] Ostrowska J, Janiszewska J, Szostak-Węgierek D (2020). Dietary acid load and Cardiometabolic risk factors—a narrative review. Nutrients.

[89] Krupp D, Esche J, Mensink GBM, Klenow S, Thamm M, Remer T (2018). Dietary acid load and potassium intake associate with blood pressure and hypertension prevalence in a representative sample of the German adult population. Nutrients.

[90] Găman MA, Epîngeac ME, Diaconu CC, Găman AM (2020). Evaluation of oxidative stress levels in obesity and diabetes by the free oxygen radical test and free oxygen radical defence assays and correlations with anthropometric and laboratory parameters. World J Diabetes.

[91] Cottone S, Mulè G, Nardi E, Vadalà A, Guarneri M, Briolotta C, Arsena R, Palermo A, Riccobene R, Cerasola G (2006). Relation of C-reactive protein to oxidative stress and to endothelial activation in essential hypertension. Am J Hypertens.

[92] Cauci S, Xodo S, Buligan C, Colaninno C, Barbina M, Barbina G, Francescato MP (2021). Oxidative stress is increased in combined oral contraceptives users and is positively associated with high-sensitivity C-reactive protein. Molecules.

[93] Gogga P, Śliwińska A, Aleksandrowicz-Wrona E, Małgorzewicz S (2019). Association between different types of plant-based diets and leptin levels in healthy volunteers. Acta Biochim Pol.

[94] Kim MH, Bae YJ (2015). Comparative study of serum leptin and insulin resistance levels between Korean postmenopausal vegetarian and non-vegetarian women. Clin Nutr Res.

[95] Wu T, Seaver P, Lemus H, Hollenbach K, Wang E, Pierce JP (2019). Associations between dietary acid load and biomarkers of inflammation and hyperglycemia in breast cancer survivors. Nutrients.

[96] Giugliano D, Ceriello A, Esposito K (2006). The effects of diet on inflammation: emphasis on the metabolic syndrome. J Am Coll Cardiol.

[97] Hribal ML, Fiorentino TV, Sesti G (2014). Role of C reactive protein (CRP) in leptin resistance. Curr Pharm Des.

[98] Weinhold B, Rüther U (1997). Interleukin-6-dependent and -independent regulation of the human C-reactive protein gene. The Biochemical journal.

[99] Sudhakar M, Silambanan S, Chandran AS, Prabhakaran AA, Ramakrishnan R (2018). C-reactive protein (CRP) and leptin receptor in obesity: binding of monomeric CRP to leptin receptor. Front Immunol.

[100] Prodam F, Filigheddu N (2014). Ghrelin gene products in acute and chronic inflammation. Arch Immunol Ther Exp.

